# Antagonistic pleiotropy at the *stem solidness 1* locus balances wheat stem architecture and yield components under terminal drought

**DOI:** 10.1007/s00122-026-05330-6

**Published:** 2026-08-16

**Authors:** Simeon Ntawuguranayo, Roy Sadeh, Hanan Sela, Ittai Herrmann, Zvi Peleg, Roi Ben-David

**Affiliations:** 1 The Robert H. Smith Faculty of Agriculture, Food, and Environment, The Hebrew University of Jerusalem, 7610001 Rehovot, Israel; 2 The Institute of Plant Sciences, Agriculture Research Organization (ARO) - Volcani Institute, 7505101 Rishon LeZion, Israel; 3 Institute of Evolution, University of Haifa, 3498838 Haifa, Israel

## Abstract

**Key message:**

Stem structural investment enhances grain size and yield under drought, challenging the traditional view of vegetative-reproductive trade-offs. Antagonistic pleiotropy at the *SSt1* locus reveals a genetic constraint between stem diameter and solidness. Incorporating genotype-by-environment into genomic selection models improves prediction accuracy by 46.5%.

**Abstract:**

Terminal drought is the primary environmental constraint on wheat productivity in the Mediterranean basin, a challenge intensifying under climate change. While stem biomass investment is known to promote grain-filling under stress, its underlying genetic architecture remains poorly understood. We evaluated a bread wheat diversity panel (*n* = 295; WheatMore) across three Mediterranean environments: well-watered (635 mm), terminal drought (228 mm), and rain-fed (468 mm). Our objectives were to characterize the phenotypic and genetic trade-offs between stem traits and yield components, identify associated genomic regions, and implement multi-environment genomic selection models. Increased investment in stem structural biomass, specifically dry weight, diameter, and peduncle length, was positively correlated with grain size and yield per spike, particularly under terminal drought. These results suggest that robust stem architecture enhances source strength without penalizing yield, as increased grain weight compensates for the biomass investment. Genome-wide association identified 80 significant SNPs and 77 gene hits, including 14 stable, 61 environment-specific, and 21 pleiotropic loci. Notably, pleiotropic regulation at the *stem solidness* *1* locus (*SSt1*) on chromosome 3B revealed a genetic trade-off between stem diameter and solidness mediated by antagonistic alleles, highlighting a constraint for simultaneous optimization. Multi-environment genomic prediction incorporating genotype-by-environment interaction outperformed single-environment models, increasing prediction accuracy by 46.5%. These findings provide breeders with markers and predictive tools to develop wheat stem ideotypes to optimize climate adaptability.

**Supplementary Information:**

The online version contains supplementary material available at https://doi.org/10.1007/s00122-026-05330-6.

## Introduction

Climate change, toward increasing environmental instability, poses significant challenges to global food security. In this context, enhancing the yields of staple grain crops, such as bread wheat (*Triticum aestivum*), is essential. The wheat stem plays a pivotal role in climate adaptability, particularly under terminal drought post-anthesis stress conditions in the Mediterranean basin. Beyond providing structural support for leaves and grain-bearing spikes, the stem facilitates the transport of water and minerals and serves as a critical reservoir for assimilates, water-soluble carbohydrates (WSC), which support grain-filling when photosynthesis is inhibited by stress (Ehdaie et al. 2006b, a; Scofield et al. 2009; Blum et al. 1997).

Recent studies have demonstrated that stem traits such as internode diameter and peduncle length are critical determinants of grain-filling capacity. These traits correlate positively with the accumulation and mobilization of WSCs, contributing significantly to grain size and yield stability under both water-limited and heat-stress conditions (Ntawuguranayo et al. 2024; Taria et al. 2025; Malakondaiah et al. 2025). Furthermore, Sallam et al. (2026) reported that peduncle weight and diameter are significantly associated with yield components across contrasting water availabilities. However, some studies document variable or non-significant correlations between stem biomass traits and grain yield across genotypes and environments (Jaradat 2009; Joudi et al. 2017; Gurumurthy et al. 2023). This inconsistency underscores the need to examine whether genomic regions governing stem traits confer consistent advantages or entail trade-offs with yield components in Mediterranean terminal drought conditions.

Despite the above-mentioned associations with yield-related traits, morphophysiological stem traits, such as diameter, peduncle length, and dry weight, have received limited attention in breeding programs. Dissecting the genetic architecture of these traits is essential for developing a wheat stem ideotype optimized for the Mediterranean climate. Genome-wide association studies (GWAS) have emerged as a powerful tool for dissecting the genetic architecture of wheat stem variation, successfully identifying quantitative trait loci (QTLs) for peduncle length (Godoy et al. 2018; Liu et al. 2023), WSC (Dong et al. 2016), stem diameter, strength, and wall thickness (Miao et al. 2025; Wang et al. 2025). Stem solidness was found to be governed by the *solid stem* (*SSt1*) locus on chromosome 3B and regulated by the *Dof-type zinc finger* transcription factor (*TdDof*; Cook et al. 2004; Nilsen et al. 2017; 2020). However, GWAS often fails to capture the collective effects of numerous small-effect QTLs underlying complex adaptive traits, particularly those exhibiting genotype × environment (*G* × *E*) interactions.

Genomic selection (GS) has been proposed to overcome these limitations by leveraging high-density genome-wide markers to estimate the genomic breeding value (GEBVs). This approach captures both major and minor QTLs, enabling effective selection for low-heritability traits influenced by strong *G* × *E* interactions (Heffner et al. 2009, 2011). By accelerating selection cycles and improving genetic gain, GS enhances the overall efficiency of developing climate-resilient varieties (Escamilla et al. 2025; Mangal et al. 2024; Jannink et al. 2010). Among GS models, genomic best linear unbiased prediction (GBLUP) is widely utilized for quantitative traits controlled by polygenic small-effect loci (Parveen et al. 2023). GBLUP has demonstrated high accuracy in predicting stem-related traits, including plant height, peduncle length, and internode diameter (Rabieyan et al. 2024; Semagn et al. 2022). Recent extensions of GBLUP, such as reaction-norm and marker-by-environment (*M* × *E*) approaches, further enhance prediction accuracy under fluctuating environmental conditions by explicitly modeling *G* × *E* interactions (Jarquín et al. 2014; Lopez-Cruz et al. 2015). Sadeh et al. (2024) and Tomar et al. (2021) emphasize that the multi-environment model consistently outperforms single-environment models, underscoring the critical need to account for environmental variation to accurately predict the genetic architecture of stem structural biomass.

The genetic control of individual stem traits, such as stem diameter, thickness, peduncle diameter, and length, has been elucidated (Wang et al. 2025; Rabieyan et al. 2024; Song et al. 2021). However, comprehensive genetic studies that integrate multiple stem traits and dissect their relationships with yield components under Mediterranean conditions remain scarce. Critically, it remains unclear whether the genomic regions governing stem traits maintain stable effects across fluctuating environmental conditions or are subject to trade-offs that limit genetic gain. In this study, we sought to test the hypothesis that a fundamental genetic trade-off exists between investment in stem structural biomass and grain yield components. Although the prevailing paradigm is that vegetative sinks usually compete with reproductive output, we investigated whether increased stem architecture actually serves as a critical physiological buffer to sustain grain size under terminal drought.

The objectives of this study were to: *i*) assess phenotypic and genetic trade-offs between investment in stem structural biomass and yield components across contrasting water availabilities; *ii*) identify genomic regions associated with stem traits; and *iii*) implement multi-environment genomic selection models for stem traits and evaluate their prediction accuracy. We demonstrated that increased investment in stem structural biomass is positively associated with grain size and yield per spike, particularly under terminal drought. Models that incorporate *G* × *E* interactions improve the predictive accuracy of stem traits, enabling efficient selection of stem ideotypes for Mediterranean environments.

## Materials and methods

### Plant material and experimental design

A diversity panel of spring bread wheat genotypes (*n* = 295; WheatMore) was used in this research. The panel was previously genotyped using the Illumina iSelect 90 K Infinium SNP array, and after filtering, 21,197 SNPs remained; full details of the genotyping are described in Sadeh et al. (2024). During the 2021–2022 season, the WheatMore panel was sown in the rain-out shelter facility at the Experimental Farm of the Hebrew University of Jerusalem, Rehovot, Israel (34°47′N, 31°54′E; 54 m above sea level) under two water regimes: well-watered (WW) and terminal drought (TD). The total cumulative water was 635 mm for WW (2022WW hereafter) and 228 mm for TD (2022TD) (Fig. S1a). The experimental design was a split-plot design with three blocks, with the water regime as the main plot and genotypes as subplots. The plot size was 80 cm long × 60 cm wide, and each plot consisted of six rows with a 10 cm spacing, at a sowing density of 220 seeds per m^2^. The full description of the experiment was previously published (Sadeh et al. 2024).

In the 2023–24 season, the panel was sown at the experimental farm of the Agriculture Research Organization at Rishon LeZion, Israel (31°59′N, 34°49′E; 40 m above sea level). An augmented experimental design with five blocks was implemented. Four commercial Israeli cultivars included in the panel (WM010, WM023, WM053, and WM299) were used as checks and were randomly assigned within each block. This environment was rain-fed (2024RF hereafter) and received a total precipitation of 468 mm (Fig. S1a). The plot size measured 7 m long × 1.6 m wide with a sowing density of 220 seeds per m^2^. The seasonal minimum and maximum temperatures for each experiment are presented in Fig. S1b.

### Phenotypic measurement

*Heading date* (HD) was determined for each plot when 50% of the spikes were fully exposed, based on daily inspections, and expressed as days after emergence (DAE). To characterize the stem traits, five random main stems from the middle rows of each plot were sampled 25 days after heading. In the 2022 experiment, due to the small plot size, only three random main stems per plot were sampled. Stem samples were separated from spikes, and lower stem internodes were separated from the peduncle and oven-dried at 60 °C for 48 h. After weighing the total sample dry weight, leaves and leaf sheaths were carefully removed from the stem and weighed to record the *stem dry weight* (SDW) of the lower internode and *peduncle dry weight* (PDW) of the last internode. *Peduncle length* (PL, uppermost internode) was measured at physiological maturity before harvesting on three main plants from the middle rows, from the beginning of the last internode to the beginning of the spike. Stem trait characterization was conducted on main stems by cutting in the middle of the second internode from the base and the last internode (peduncle). *Stem diameter* (SD) and *stem wall width* (SWW), *peduncle diameter* (PD), and *peduncle wall width* (PWW) were measured using a digital caliper (Mitutoyo Calipers, USA). The stem solidness (SS) and *peduncle solidness* (PS) were calculated as previously described (Ntawuguranayo et al. 2024).

Two samples of 30 cm each were manually harvested from the central rows of each plot to assess the yield components. The samples were weighed to determine the total above-ground *biomass* (BM). Spikes were separated from the rest of the plant’s parts and counted to determine the *spike number* (SN). Spikes were threshed using a laboratory thresher (LD350; Wintesteiger) to determine *grain yield* (GY) and *harvest index* (HI = GY/BM). For the 2024 experiment, each plot was mechanically harvested using a combiner harvester (Wintersteiger Quantum Pro) to determine GY. *Grain yield per spike* (GYPS = GY/SN). *Thousand-grain weight* (TGW) was calculated for each plot by counting 1000 seeds (Seed Counter S-25, Data Technologies) and *grain number* (GN) = (GYPS × SN)/(TGW × 1000).

### Statistical analyses

The JMP® version 19.0 pro statistical package (SAS Institute, Cary, NC) and R software 4.4.3 (https://www.r-project.org, R Core Team 2024) were used for all statistical analyses. Descriptive statistics were performed on the full dataset to examine variable distributions and to calculate the coefficient of variance (CV). A two-way analysis of variance (ANOVA) was conducted to evaluate the potential impact of genotype, environment, and genotype × environment interaction on measured traits. The mean comparison was performed using the Tukey HSD test, and linear (Pearson) correlation analyses were conducted to assess the relationship among phenotypic traits. For 2022WW and 2022TD environments, the best linear unbiased estimates (BLUEs) of genotype means for each trait were calculated using the lme4 R package (Bates et al. 2015). The BLUE values were estimated using a mixed linear model with genotypes as fixed effects and block as a random effect following the procedure described in Sadeh et al. (2024). For the 2024RF environment, the adjusted mean for all traits was computed using the AugmentedRCBD R package (Aravind et al. 2026). The genomic correlations between measured traits were calculated using the BGLR R package (Pérez and de los Campos 2014). The broad sense of heritability (_bs_*h*^2^) was calculated based on the data from the 2022 experiments and was estimated using ANOVA-based variance components:

$$_{{{\mathrm{bs}}}} h^{2} = \frac{{\sigma_{G}^{2} }}{{\sigma_{G}^{2} + \frac{{\sigma_{{{\mathrm{GE}}}}^{2} }}{{n_{E} }} + \frac{{\sigma_{ \in }^{2} }}{{n_{E} n_{r} }}}}$$

where σ^2^_G_, σ^2^_GE_, and σ^2^ϵ are the genetic, genotype-by-environment interaction, and residual variances, respectively, *n*_*E*_ is the number of environments (2), and *n*_*r*_ is the number of replicates in each environment (3).

### Genome-wide association studies

Phenotypic and genotypic data comprising 295 genotypes and 21,197 SNPs were analyzed using the Genome Association and Prediction Integrated Tool (GAPIT3) genetics statistical package (Wang and Zhang 2021) in R (R Core Team 2024). The association was analyzed using three GWAS models: mixed linear model (MLM) (Yu et al. 2006), fixed and random model, circulating probability unification (FarmCPU), and Bayesian information and linkage disequilibrium iteratively nested keyway (BLINK). These models have been used to identify both small and large effect markers and are also effective for evaluating large datasets while reducing false-positive and false-negative results (Merrick et al. 2022).

GWAS was conducted independently for each environment using the BLUE values of the 2022 environment and the adjusted mean in 2024RF. To account for multiple testing and linkage disequilibrium among 21,197 SNPs, the genome-wide significance threshold was determined using the effective number of independent tests (M_eff_) method, proposed by Li and Ji (2005). The threshold was calculated as *P* = 1 − (1 − 0.05) ^ (1/M_eff_), where 0.05 is the desired significance level, resulting in a value of (*P* = 1.96 × 10^−5^). Significant marker-trait associations (MTAs) from the three models were integrated using a union approach, in which a SNP reached the significant threshold in at least one model. False positives were controlled through allele-specific effect validation using *t*-test and examining QQ plots to confirm adequate control of genomic inflation. Circular and rectangular Manhattan plots were created using the "CMplot" R package (https://github.com/YinLiLin/CMplot; Yin et al. 2021). Pairwise linkage disequilibrium (LD) among SNP markers was assessed using the genetic and LDheatmap packages (Shin et al. 2006), where all pairwise LD values were quantified using the squared Pearson’s correlation coefficient (*r*^2^) between allele frequencies at each marker pair. A graphical representation of the genetic physical map was drawn using MapChart software v2.32 (Voorrips 2002). LD decay and marker resolution for the WheatMore panel were previously characterized by Sadeh et al. (2025), where genome-wide LD dropped to half its initial value at 56,349 base pairs. Putative candidate genes underlying significant MTAs were identified using a positional approach based on the International Wheat Genome Sequencing Consortium (IWGSC) RefSeq v1.1 assembly (Appels et al. 2018).

For each significant SNP, the corresponding sequence was queried against the RefSeq v1.1 high confidence gene set via the basic local alignment search tool (BLAST) through Ensembl Plants (https://plants.ensembl.org/Triticum_aestivum/Info/Index). Gene hits were retained only if located on the same chromosome, with sequence percent identity > 95%, and an E-value < 1e-10 to exclude homoeologous cross-mapping in the hexaploid wheat genome. Putative gene hits were defined as those directly overlapping or immediately flanking the significant SNP position. Protein functions of identified gene hits were further verified using the UniProt database (https://www.uniprot.org).

### Genomic prediction

A single-environment (SE) analysis for each trait was conducted in each environment separately using genomic best unbiased prediction (GBLUP; VanRaden 2008) and was used as a baseline for multi-environment (ME) models. The SE model was done following the mixed linear model formula: $y = {\upmu }1 + Z_{{\mathrm{g}}} g + \in$, where y is the response vector of the phenotypes, 1 is the vector of ones of orders, µ is the overall mean of the environment, *Z*_g_ is the incidence matrix for the random effect, g ∼ *N* (0, Gσ^2^_*u*_) is the vector of random genotypic effect, ϵ ∼ *N* (0, Iσ^2^ϵ) is the random residual effect, *I* is the identity matrix; σ^2^_*u*_ is the genetic variance; and σ^2^ϵ is the residual variance. *G* is the genomic relationship matrix defined as $G= \frac{\mathrm{W}\mathrm{W}{\prime}}{m}$ according to VanRaden (2008), where W is a centered and standardized gene content matrix, and m is the total number of SNP markers. The multi-environment analysis was conducted using the marker-by-environment interaction (*M* × *E*) model by Lopez-Cruz et al. (2015). The *M* × *E* model’s core concept is to partition the total genetic effects of markers into the main genetic effects across all environments and the specific genetic effects for each environment. The *M* × *E* model was described in detail by Sadeh et al. (2024).

### Assessment of prediction accuracy and cross-validation schemes

The prediction accuracy (PA) was estimated as the Pearson’s correlation coefficient between the observed phenotype and GEBV values in the testing set, representing the average Pearson’s correlation across all cross-validation (CV) iterations. This approach is used to present the PA of the models. For the single-environment model, 50 repeated subsamples were conducted in each environment, utilizing 70% of the data as the training set and 30% as the testing set, as described by Cuevas et al. (2017) and Sadeh et al. (2024). The PA of the multi-environment models was evaluated through three cross-validation scenarios (CV0, CV1, and CV2), which highlight different challenges in plant breeding (Fig. S2). The first scenario (CV0), or leave-one-environment-out, used data from all lines from two environments to predict their breeding values in a new environment. The second scenario (CV1) assessed the ability to predict the breeding values of new lines that have not been phenotyped in any environment, relying on known lines with phenotypic data from all three environments. The third scenario (CV2) evaluated the ability to predict the breeding values of lines that were grown in some environments but not in others, with the training population containing phenotypic data from all environments and the test population including lines with missing data from one randomly selected environment.

## Results

### High phenotypic diversity was found in the WheatMore panel

**Fig. 1:**
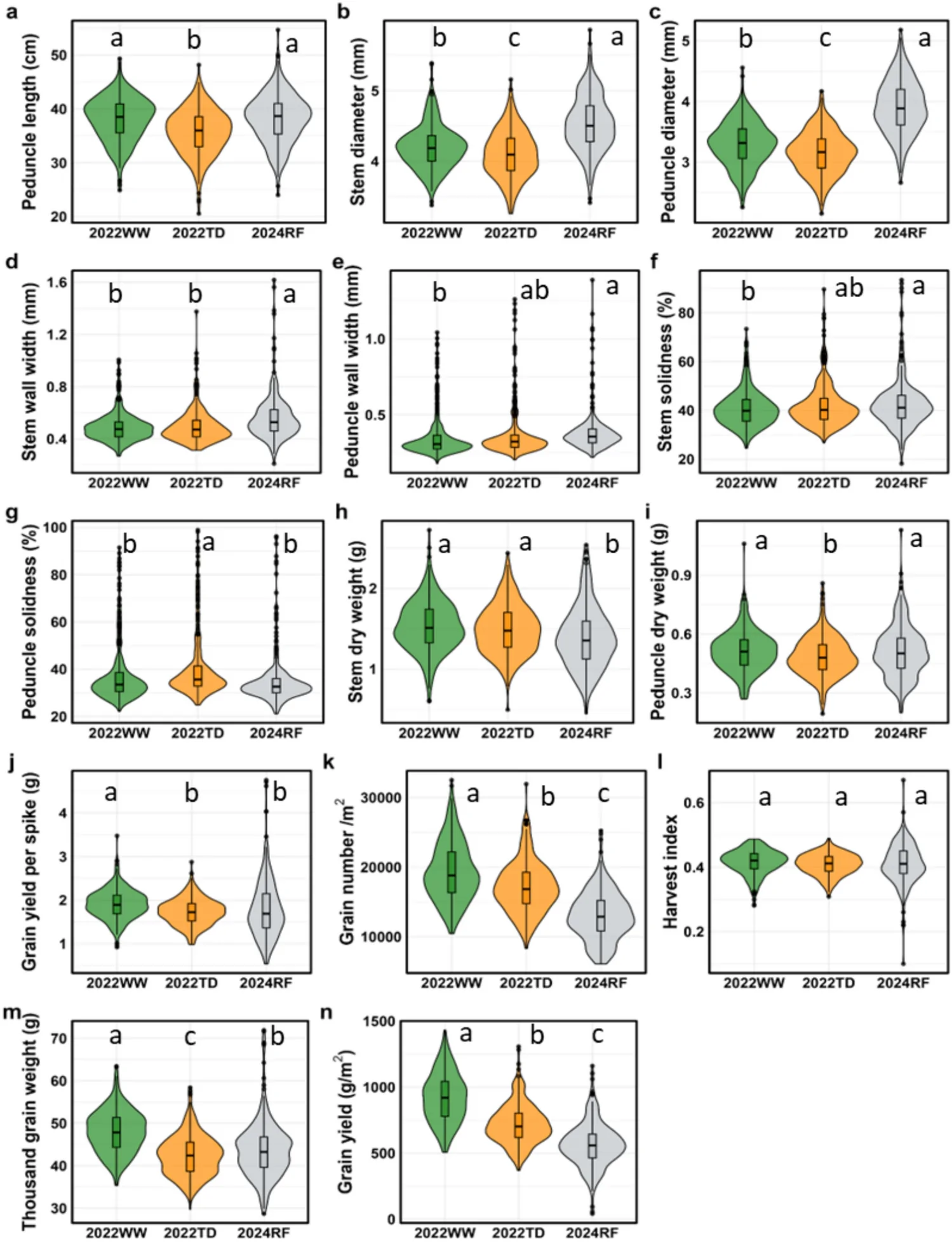
The WheatMore diversity panel phenotypic characterization across the three environments. Violin plots of measured stem traits and yield components of the panel in three environments: well-watered (2022WW), terminal drought (2022TD), and rain-fed (2024RF). **a** peduncle length, **b** stem diameter, **c** peduncle diameter, **d** stem wall width, **e** peduncle wall width, **f** stem solidness, **g** peduncle solidness, **h** individual stem dry weight, **i** individual peduncle dry weight, **j** grain yield per spike, **k** grain number, **l** harvest index, **m** thousand-grain weight, and **n** grain yield. Different letters indicate a significant difference between environments (*P* ≤ 0.05). Environments 2022WW and 2022TD had three replicates, and the 2024RF used an augmented design (colour figure online)

To assess the phenotypic diversity within the WheatMore panel, we characterized the wheat genotypes under Mediterranean basin conditions in three environments: well-watered (2022WW), terminal drought (2022TD), and rain-fed (2024RF). In general, the WheatMore panel exhibited rich variation for stem traits and yield components with significant environmental effects (Fig. 1). The mean peduncle length ranged from 35 cm under 2022TD to 38 cm under 2022WW and 2024RF, with high heritability (_bs_*h*^2^ = 0.82; Fig. 1a, Table S1).

Under TD, plants exhibited narrower stem diameters at both the second internode and the peduncle compared to other environments (Fig. 1b, c). Mean peduncle diameter showed a more pronounced environmental response, decreasing from 3.92 mm in 2024RF to 3.15 mm under 2022TD (Table S2). Mean stem wall width, solidness, and PWW were higher in 2024RF, with no significant differences observed between the 2022WW and 2022TD for these traits (Fig. 1d–f). In contrast, peduncle solidness was higher under TD (41%), while no difference was observed between WW (38%) and rain-fed (35%) (Fig. 1g, Table S2).

Biomass allocation to the stem varied among environments. Individual stem DW mean was lowest in 2024RF (1.39 g), with similar values observed in 2022TD and 2022WW (1.49 and 1.52 g, respectively). Individual peduncle DW mean ranged from 0.48 g in 2022TD to 0.51 g in both 2022WW and 2024RF (Fig. 1h, i, Table S2). The heritability estimates for stem traits were moderate to high, with stem DW showing the lowest heritability (_bs_*h*^2^ = 0.67, Table S1). Yield components were highly affected by environmental conditions, except for harvest index (Fig. 1j–n).

To assess genotype × environment interactions, we used analysis of variance (for the 2022 experiment), resulting in no significant *G* × *E* interactions for most stem traits, except for stem wall width (*P* = 0.05) and stem solidness (*P* = 0.02). In contrast, yield components showed significant *G* × *E* interactions, except grain yield per spike (*P* = 0.47, Table S1).

### A strong phenotypic and genomic correlation between stem and peduncle dry weight, grain size, and yield per spike

**Fig. 2:**
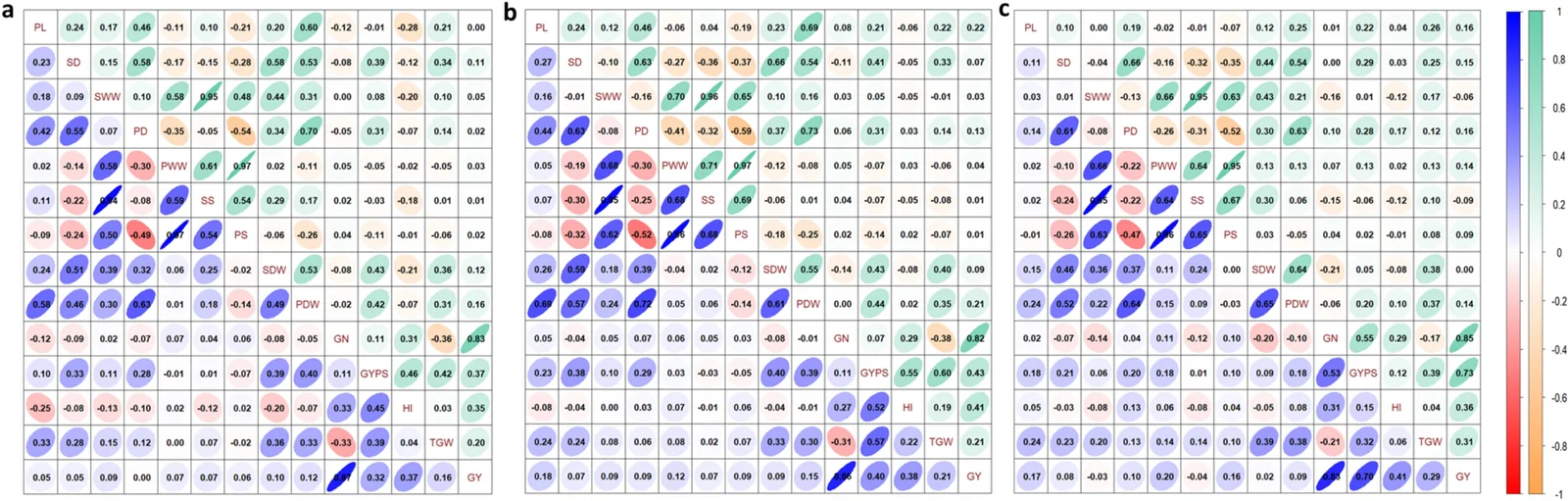
Phenotypic and genomic correlation between measured traits under **a** well-watered (2022WW), **b** terminal drought (2022TD), and **c** rain-fed (2024RF). Pearson’s correlation matrix (lower palette of the diagonal in red-blue scale) and the heat map of genomic correlation (upper palette of the diagonal, orange-cyan scale) between measured traits: peduncle length (PL); stem diameter (SD); stem wall width (SWW); peduncle diameter (PD); peduncle wall with (PWW); stem solidness (SS); peduncle solidness (PS); individual stem dry weight (SDW); individual peduncle dry weight (PDW); grain number (GN); grain yield per spike (GYPS); harvest index (HI); thousand-grain weight (TGW); and grain yield (GY) per square meter. Significant phenotypic correlations are presented in Table S3 (colour figure online)

Phenotypic and genomic correlations among all measured traits were computed within each environment to examine relationships between stem morpho-physiological traits and yield components. Genomic correlations (*r*_g_) were slightly higher than phenotypic correlations (*r*_*p*_). Individual stem DW exhibited the highest genomic and phenotypic correlations with TGW across all environments (*r*_g_ = 0.36, 0.40, and 0.38, and *r*_*p*_ = 0.36, 0.33, and 0.39, *P* < 0.0001, under 2022WW, 2022TD, and 2024RF, respectively; Fig. 2). A similar pattern was found between PDW and TGW (Fig. 2, Table S3).

Importantly, SDW and PDW also demonstrated both positive genomic and phenotypic correlations with grain yield per spike (GYPS) across environments. In both terminal drought and well-watered environments, SDW correlated with GYPS (*r*_g_ = 0.43), while no correlation was observed in the rain-fed environment. Similarly, PDW showed positive genomic correlations with GYPS across all three environments (*r*_g_ = 0.42, 0.44, and 0.20 under 2022WW, 2022TD, and 2024RF, respectively). The phenotypic correlations between SDW and PDW with GYPS followed a similar trend, with the highest phenotypic correlation observed under terminal-drought conditions (r_*p*_ = 0.40, *P* < 0.0001 for SDW; and *r*_*p*_ = 0.39, *P* < 0.0001 for PDW) (Fig. 2b, Table S2).

Stem and peduncle diameter also contributed positively to grain size and GYPS under 2022TD and 2022WW. However, these correlations were weaker in a rain-fed environment than in well-watered and terminal drought (Fig. 2, Table S3). Stem and peduncle diameter were associated with biomass accumulation. SD and PD were strongly associated with their respective dry weights. PD consistently exhibited higher genomic correlations with individual PDW (*r*_*g*_ = 0.73, 0.70, 0.63, and *r*_*p*_ = 0.72, 0.63, 0.64, *P* < 0.0001, under 2022TD, 2022WW, and 2024RF, respectively; Fig. 2, Table S3). A similar trend was observed between stem diameter and stem DW across all environments.

Negative genomic and phenotypic correlations between SD and SS, as well as between PD and PS, revealed fundamental trade-offs in wheat stem architecture. PD showed a strong negative association with PS across all environments (r_*g*_ = − 0.58, − 0.55, − 0.51, and r_*p*_ = − 0.52, − 0.49, − 0.47; *P* < 0.0001, under 2022TD, 2022WW, and 2024RF, respectively. SD exhibited moderate negative genomic correlations with SS (r_*g*_ = − 0.33, − 0.16, − 0.31, and r_*p*_ = − 0.30, *P* < 0.0001; − 0.22, *P* = 0.0001; − 0.24, *P* = 0.0003, under 2022TD, 2022WW, and 2024RF, respectively; Fig. 2, Table S3).

### Genome-wide association analysis identified adaptive, stable markers, and gene hits for stem traits

We used a multi-model GWAS approach (MLM, BLINK, and FarmCPU) to enhance our ability to detect both small and large effect MTAs across different genetic architectures and effect sizes. About 80 significant SNPs were associated with PL, SD, SWW, SS, PD, PWW, PS, SDW, and PDW. Among them, 14 SNPs were stable (consistent) across all three environments, while 61 SNPs were environment-specific, with 26, 20, and 15 under 2022TD, 2022WW, and 2024RF, respectively. Additionally, five SNPs were detected across two environments, and 21 SNPs exhibited pleiotropic effects, concentrated on chromosomes (chr.) 3B, 3D, 5A, 6A, and 6B.

In total, 77 putative candidate genes were identified using IWGSC RefSeq v1.1 and UniProt annotations, showing diverse functional roles relevant to stem cell architecture and structural development, including oxidoreductase activity, nucleotide binding, metal ion binding, transmembrane transport, and cell wall organization. The list of all identified SNPs, gene hits, and environment-specific results is presented in Table S4 and Fig. S3, with functional annotations in Table S5.

*Peduncle length:* Five adaptive SNPs (-log10_(*P*)_ 4.92-8.61) were identified. Under 2022TD, marker *Kukri_c29110_360* (6A, 5.22% PVE) was linked with *TraesCS6A02G074200* (oxidoreductase activity). The SNP marker *BS00023043_51* on chr. 2B (4.22% PVE) was identified in 2022WW and linked to *TraesCS2B02G069000* (transmembrane transport protein, ATP hydrolysis). In 2024RF, three markers: *RAC875_c47696_532* (5B)*, RFL_Contig897_207* (6B)*,* and *Tdurum_contig26316_301* (7B)*,* each explaining approximately 6.7% PVE, were associated with genes involved in oxidoreductase and DNA binding activities (Tables S4 and S5).

**Fig. 3:**
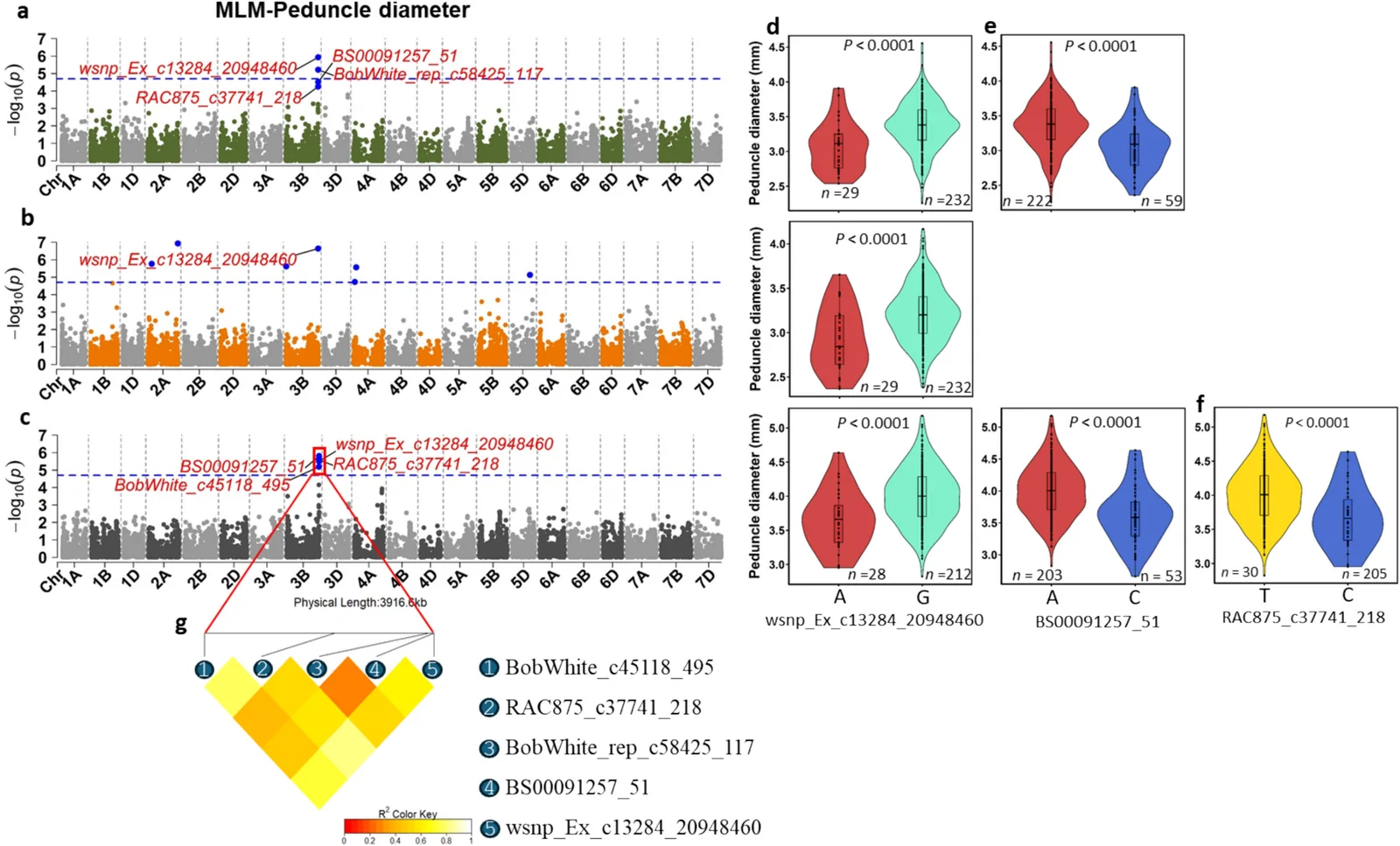
Genome-wide association studies of peduncle diameter. Manhattan and violin plots of peduncle diameter. Manhattan plot of peduncle diameter in **a** well-watered (2022WW), **b** terminal drought (2022TD), and **c** rain-fed (2024RF) environments. Each point in the Manhattan plot represents a single genetic marker along the genome. A dashed blue line indicates the significance threshold, while significantly associated markers are highlighted in blue. **d** significant marker identified in three environments; **e** significant marker in 2022WW and 2024RF; **f** significant marker identified in 2024RF. **g** Linkage disequilibrium (LD) plot for the significant marker associated with peduncle diameter on chromosome 3B, where R^2^ color scale indicates the level of linkage disequilibrium from low (red) to high (yellow). The number of genotypes (*n*) is indicated next to each violin. The *P*-value indicates the difference between the favorable and alternative alleles using a *t-*test (colour figure online)

*Stem and peduncle diameter:* A total of 23 SNPs were associated with SD and PD. For SD, the SNP marker *Kukri_rep_c105296_156* (6A) was consistently detected under both well-watered and rain-fed conditions, contributing 9.51–11.73% PVE. The SNP marker *Tdurum_contig27982_568* (3B, 8.10% PVE) was adaptive under WW (Table S4). For PD, 16 SNPs were identified across three environments, with *wsnp_Ex_c13284_20948460* (3B) being the most stable SNP, contributing 7.12–13.07% PVE across all three environments (Fig. 3a–c). A pleiotropic marker, *BobWhite_c45118_495* (3B), was dually associated with both SD (9.51% PVE) and PD (11.62% PVE) under rain-fed conditions. Its corresponding gene hit, *TraesCS3B02G595200,* encodes a ubiquitin-protein ligase with zinc ion binding activity and was additionally associated with all four solidness traits (SWW, PWW, SS, and PS) across all three environments, indicating its potential role in coordinating both stem diameter and solidness.

*Stem wall width and solidness:* Sixteen SNPs (1.43 − 21.16% PVE) for SWW, and 20 SNPs (2.58 − 21.69% PVE) for SS were identified on chrs. 1A, 2A, 2D, 3B, and 3D. Three stable SNPs on chr. 3B (*BS00091257_51, RAC875_c37741_218, wsnp_Ex_c13284_20948460*) were jointly associated with both traits (Table S4). Gene hits for these solidness traits include 38 putative genes, of which 13 were consecutively detected in two or three environments, and 25 were environment-specific and responsive to water availability (Table S5).

**Fig. 4:**
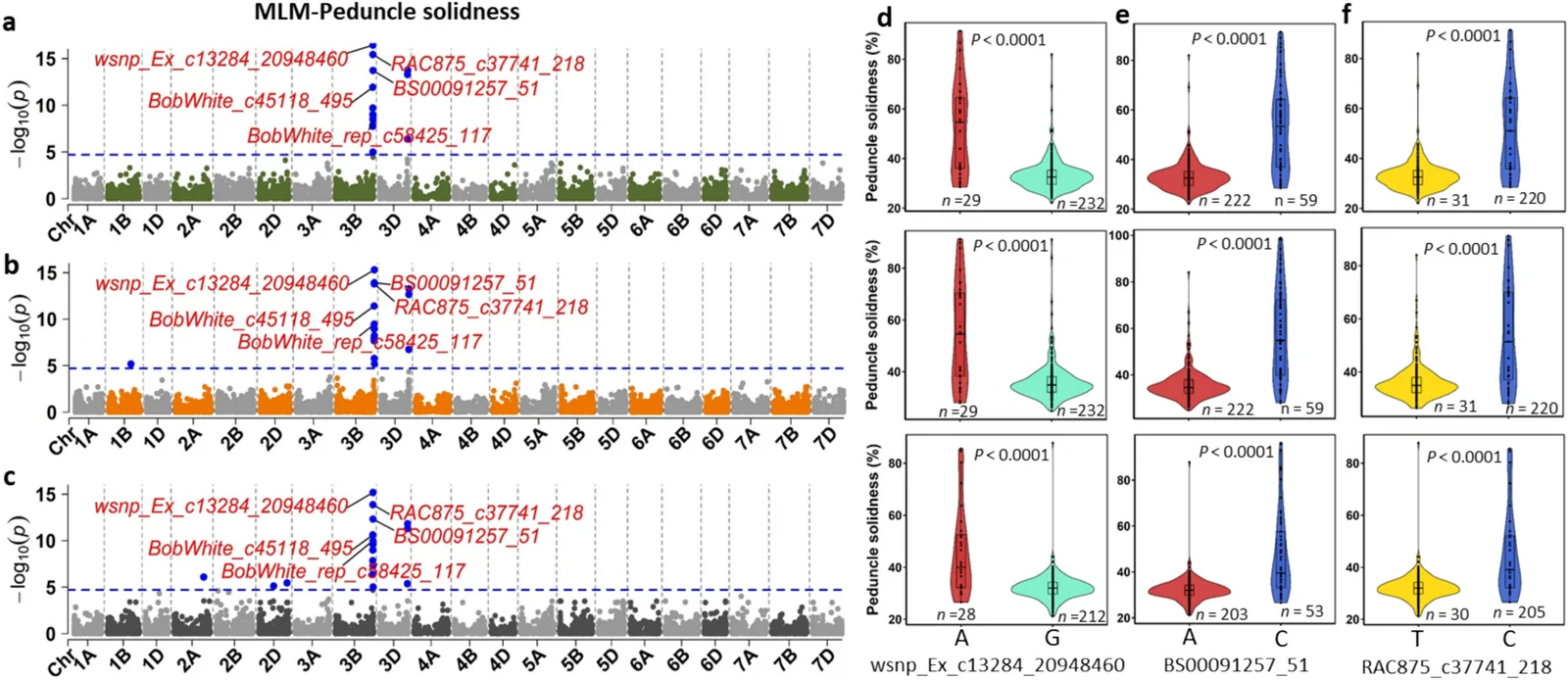
Genome-wide association studies of peduncle solidness. Manhattan and violin plots of peduncle solidness. Manhattan plots for peduncle solidness under **a** well-watered (2022WW), **b** terminal drought (2022TD), and **c** rain-fed (2024RF) environment. Each point in the Manhattan plot represents a single marker along the genome. A dashed blue line indicates the significance threshold, while significant associated markers are highlighted in blue. **d-f** Violin plots of significant markers identified in three environments. The number of genotypes (*n*) is indicated next to each violin. The *P*-value indicates the difference between the favorable and alternative alleles using a *t-*test (colour figure online)

*Peduncle wall width and peduncle solidness:* Strong genomic signals were found for PWW and PS, with a major cluster of ten highly significant markers on chr. 3B (818.3–825.3 Mbp) explaining15.10–40.82% PVE across all three environments. Three additional SNPs (*Excalibur_c4803_830, BS00065603_51,* and *BS00068415_51*) on chr. 3D (607.9–612.9 Mbp) were linked to both traits across three environments and accounted for 13.58–33.81% PVE (Fig. 4a–c, Table S4). Fourteen of 26 PWW MTAs and 13 of 22 PS MTAs were stable, with 12 SNPs common to both traits. Eleven gene hits were identified within a 7 Mbp interval (818.3–825.3 Mbp on chr. 3B. On chr. 3D within 5 Mbp (607.9–612.9 Mbp), three genes (*TraesCS3D02G531700, TraesCS3D02G539600,* and *TraesCS3D02G541800*) involved in intracellular signal transduction were constitutively associated with PWW and PS across all environments.

*Stem and peduncle dry weight:* Seventeen SNPs linked to SDW were identified under 2022TD and 2022WW, with *Kukri_c33668_877* (6D) observed in both environments. Three SNPs associated with PDW under 2022WW were located on chrs. 2B, 5A, and 6A explaining (2.04–10.65% PVE). Additional adaptive SDW markers were detected under WW conditions on chromosomes 3A, 5A, and 7A, while 10 SNPs identified in 2022TD spanned chrs. 1A, 3A, 3B, 5A, 6A, 7A, and 7B, explaining 2.01% to 12.02% (Table S4). Seventeen gene hits were linked to SDW and PDW, with a single consecutive gene*, TraesCS6D02G087200* (6D), associated with *Kukri_c33668_877*, detected under both terminal drought and well-watered environments. This gene encodes a protein with zinc ion binding activity involved in DNA double-strand break repair via homologous recombination (Table S5).

### Antagonistic pleiotropy of stem architecture at the *stem solidness 1* (*SSt1*) locus

We identified 12 SNP markers within the major stem solidness locus *SSt1* (818.3–825.3 Mbp) on chr. 3B (Fig. S4a) that are significantly associated with multiple stem traits, including stem, peduncle solidness, diameter, and wall width. These results extend the phenotypic scope of the *SSt1* region beyond stem solidness alone, indicating pleiotropic control of coordinated stem architecture. A genetic trade-off between diameter and solidness was identified, with antagonistic allelic effects at key loci associated with both traits. The SNP marker *wsnp_Ex_c13284_20948460* (chr. 3B) was identified in all three environments and associated with five traits (PD, SWW, SS, PWW, and PS).

The guanine (G) allele for this SNP is associated with increased peduncle diameter (Fig. 3d), whereas the adenine (A) allele favors peduncle solidness (Fig. 4d), demonstrating a direct allelic trade-off. Similarly, the SNP marker *BS00091257_51* (3B), identified in 2022WW and 2024RF for PD, and across three environments for PS, PWW, SS, and SWW, exhibited contrasting favorable alleles: adenine (A) for peduncle diameter (Fig. 3e) and cytosine (C) for peduncle solidness (Fig. 4e) and other solidness-related traits (PWW, SS, and SWW, Table S4). Three additional highly significant markers on chr 3B: *RAC875_c37741_218* (Figs. 3f, 4f), *BobWhite_rep_c58425_117*, and the marker *BobWhite_c45118_495* (Table S4) reinforced this pattern. The five markers displaying antagonistic allelic effects formed a block of linkage disequilibrium (Fig. 3g). Collectively, within the proximal *SSt1* interval on chr. 3B (818.3–825.3 Mbp), we identified 11 gene hits associated with multiple stem and peduncle traits across three environments (Fig. S4b).

Notably, the gene *TraesCS3B02G60220*, which was mapped to ~ 822.19 Mbp, showed consistent pleiotropic effects and emerged as the most environmentally stable candidate, identified consistently across all tested environments. This gene co-localized with our two peak marker associations on chr. 3B: *wsnp_Ex_c13284_20948460,* associated with five traits (PD, SWW, PWW, SS, and PS) across all environments, and *BS00091257_51,* which shared association under well-watered and rain-fed conditions for PD, as well as with four solidness traits (SWW, PWW, SS, and PS) under three environmental conditions (Table S5).

### Genotype × environment interactions improve stem trait prediction accuracy

**Fig. 5:**
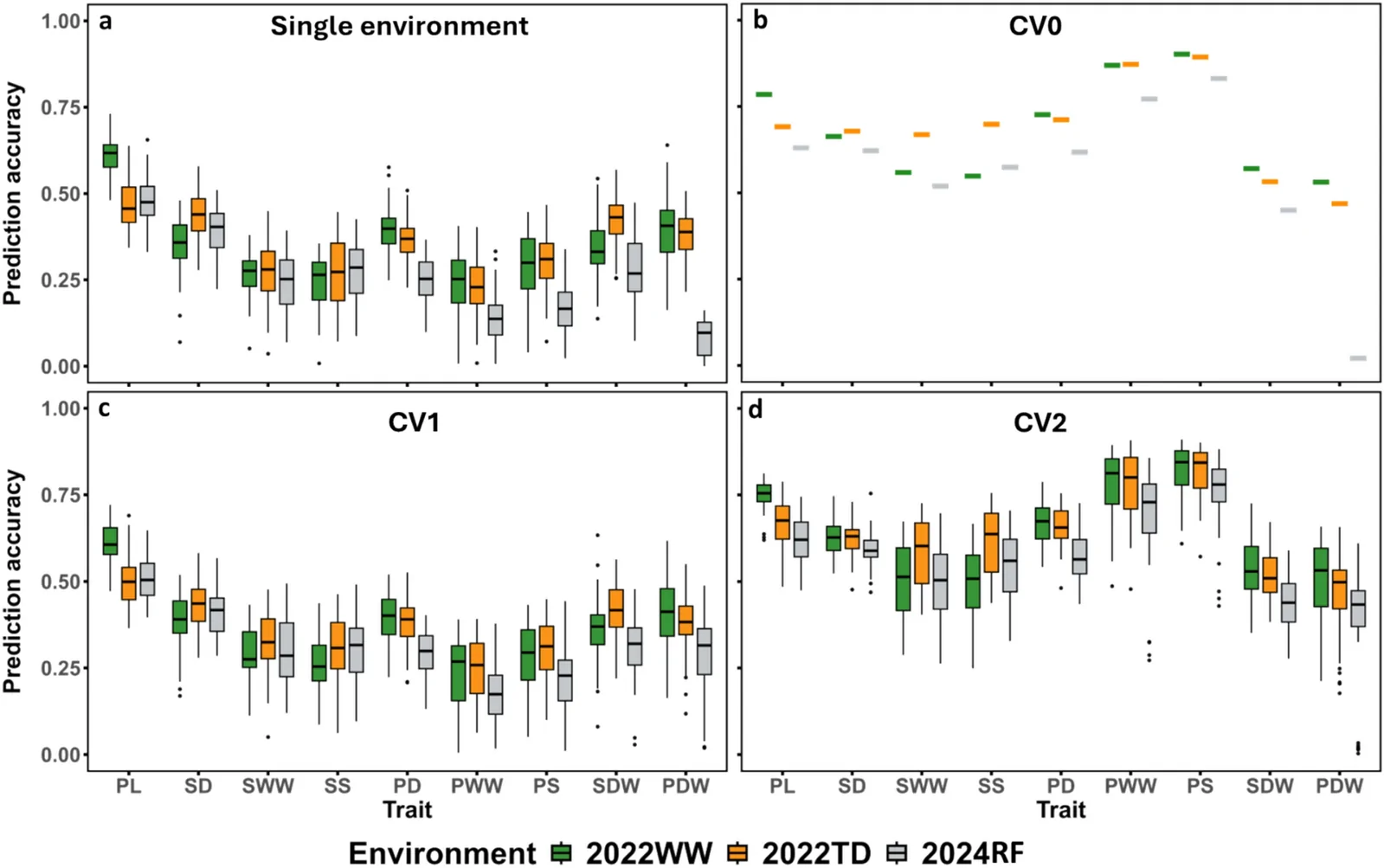
Prediction accuracy across different cross-validation scenarios. **a** Single-environment and multi-environment cross-validation scenarios; **b** CV0, **c** CV1, **d** CV2 for the predicted stem traits: peduncle length (PL), stem diameter (SD), stem wall width (SWW), stem solidness (SS), peduncle diameter (PD), peduncle wall with (PWW), peduncle solidness (PS), individual stem dry weight (SDW), and individual peduncle dry weight (PDW). Colors indicate prediction accuracy in different environments: green (2022WW, well-watered), orange (2022TD, terminal drought), and gray (2024RF, rain-fed) (colour figure online)

The prediction accuracy of nine stem traits was evaluated using single-environment (SE) and multi-environment cross-validation (CV) scenarios. Single-environment PAs varied among traits and environments. PL showed the highest prediction accuracy, with mean values of 0.61, 0.48, and 0.47, followed by SD with the PAs of 0.35, 0.44, and 0.39 under 2022WW, 2022TD, and 2024RF, respectively. The lowest single-environment PA was observed in 2024RF for PDW (0.09), PWW (0.14), and PS (0.17) (Fig. 5a). Multi-environment models substantially improved PA compared to SE models, and high-heritability traits showed the highest gain in PA across all CV scenarios.

In the CV0 scenario (Fig. 5b), which involved leaving one-environment-out while using all lines from the other two environments to predict their breeding value in a third environment (new environment). Traits with high _bs_*h*^2^ (e.g., PS, PWW, PL, and PD) showed the greatest improvements. For example, PS (_bs_*h*^2^ = 0.93) achieved the PA of 0.89, 0.90, and 0.83, compared with SE PAs of 0.30, 0.29, and 0.17 under 2022TD, 2022WW, and 2024RF, respectively. Similar relative gains were observed for other traits with moderate and low heritability.

In the CV1 scenario (Fig. 5c), which predicts breeding values for new lines that have not been phenotyped in any environment, using known lines with data in all environments. The PAs in CV1 for most of the traits were nearly identical or slightly higher than those observed in SE (Table S6). The most significant improvement in prediction accuracy was observed for PDW in 2024RF, where CV1 achieved a PA of 0.29 compared to 0.09 in SE (*P* < 0.0001; Table S6).

For the CV2 scenario (Fig. 5d), which evaluates breeding values for lines with data in some environments but not in others, the PAs for all traits were comparable to CV0 but significantly higher than those observed in SE (*P* < 0.0001; Table S6). Peduncle solidness, wall width, and peduncle length exhibited the highest PA in the CV2 scenario. PDW had the lowest PA of 0.46, 0.50, and 0.38 in CV2; however, these values were significantly higher than the corresponding PAs in SE with 0.38, 0.40, and 0.09 under 2022TD, 2022WW, and 2024RF, respectively (Table S6). Most traits (PD, PWW, PS, SDW, and PDW) were predicted more accurately in 2022WW and 2022TD environments compared to 2024RF across all cross-validation scenarios.

## Discussion

Developing wheat varieties that sustain high grain yield under a changing climate is essential for maintaining global food security. Historical breeding programs prioritized grain yield by increasing HI, mainly through reduced plant height, which shifted assimilate partitioning toward grains at the expense of vegetative organs such as the stem (Acreche et al. 2008). Within this paradigm, stem morpho-physiological traits (diameter, peduncle length, and dry weight) were largely overlooked, assuming that greater structural biomass would necessarily trade-off with GY (Rivera-Amado et al. 2019). Exploring the genetic architecture of stem traits and their relationships with yield components is therefore critical for testing this long-standing trade-off hypothesis and for elucidating mechanisms that mitigate yield loss from terminal drought. Recent studies demonstrate the role of the stem traits in enhancing wheat’s climate resilience (Taria et al. 2025; Malakondaiah et al. 2025; Ntawuguranayo et al. 2024). Our results show that increased investment in stem structural biomass does not inherently reduce grain yield; rather, the gain in TGW associated with higher stem and peduncle dry weight was sufficient to sustain, and in some cases improve, GY per spike under  terminal drought.

Our findings challenge the traditional trade-off view and highlight the stem ideotype that enhances source strength under stress. Additionally, we demonstrate that the integrated application of GWAS and GS, alongside advanced *G* × *E* modeling, provides a powerful framework valuable for dissecting the genetic basis of stem traits and accelerating the development of wheat stem ideotypes suitable for a changing climate.

### Stem structural biomass: A strategic reservoir for yield stability under terminal drought

The post-anthesis grain-filling phase coincides with terminal drought stress, shifting the source-sink balance. Stem dry weight showed a positive correlation with directly measured WSC across different environments (Ntawuguranayo et al. 2024; 2026; Talukder et al. 2013; Ehdaie et al. 2006a, b; 2008). Based on these well-established correlations, here, we used SDW and PDW as integrative proxies for carbon investment in the stem reserve. Our findings demonstrate that stem structural biomass, specifically dry weight and diameter, serves as a critical physiological buffer against stress-induced photosynthetic decline (Mostafaie et al. 2025; Vaezi et al. 2024; Ntawuguranayo et al. 2024, 2026). We observed consistent phenotypic and genomic correlation between investment in stem structural biomass, especially SDW and PDW, with TGW across all tested environments. Importantly, SDW and PDW were consistently associated with GYPS under terminal drought but not under rain-fed conditions (Fig. 2, Table S3). Similarly, Sallam et al. (2026, 2015) demonstrated that stem and peduncle dry weight are significantly associated with spike productivity traits under different environmental conditions.

In the current study, SD and PD were strongly associated with their respective dry weight and positively contributed to grain size and yield, with the strongest effects observed under stress (Fig. 2, Table S3). Plants with wider stems and longer peduncles have greater storage capacity for WSCs, enabling sustained assimilate remobilization to the developing grain mass under stress conditions (Ntawuguranayo et al. 2024, 2026; Kong et al. 2010). Fang et al. (2024) further showed that the contribution of remobilized pre-anthesis assimilates to grain biomass increases with stress intensity, ranging from nil to 100%, with water deficit having a greater impact than temperature alone. These findings underline stem traits as a possible breeding target that enhances assimilate reservoirs to improve source strength. The lack of correlation between stem DW and GYPS under rain-fed conditions (neither positive nor negative) highlights the environment-dependent nature of this trait-trait relationship (Joudi et al. 2017; Ma et al. 2025).

It is also important to note that investment in stem structural biomass did not entail a trade-off with overall grain yield across environments, as the increase in TGW associated with higher stem and PDW compensated for the biomass investment and enhanced GYPS most clearly under TD conditions. Overall, selecting for increased stem and PDW through wider stems, peduncle diameter, and longer peduncles can enhance assimilate transport to the developing grain and provide a pathway to increase grain size and yield, particularly under drought conditions. Our study not only highlights the importance of integrating such selection into pre-breeding programs but also suggests minimal risk of reduced wheat yield potential.

### Multi-environment genome-wide association analysis revealed pleiotropic effects of the *SSt1* region, extending its known role beyond stem solidness

The major finding of the current study is the identification of a pleiotropic QTL on chr. 3B (818.3–825.3Mbp; Fig. S4a) containing 12 SNP markers within a 7.1Mbp interval. This region explained up to 40.82% of the variation in PWW and PS, 19.06% for SWW and SS, 9.51% for SD, and 13.07% for PD across three environments (Table S4). This QTL co- localizes with the previously identified *Qss.msub-3BL* (*SSt1*) locus, which reportedly contributed around 76% of the variation in stem solidness in hexaploid winter wheat (Cook et al. 2004), 77.8% in spring wheat, and 92% in durum wheat (Nilsen et al. 2017).

Nilsen et al. (2020) identified a Dof zinc finger transcription factor, *TdDof* (DNA binding with one finger protein) within the *SSt1* locus as the causal gene that determines stem solidness phenotype in durum wheat, corresponding to *TraesCS3B01G60880* at approximately 828.1Mbp on chromosome 3B of the IWGSC RefSeq v1.1 assembly. However, its effect on other stem traits was not mentioned. In our study, the 12 *SSt1* SNPs and their 11 gene hits associated with multiple stem and peduncle traits are located ~ 3–10 Mbp upstream of *TdDof* within 818.3–825.3 Mbp (Fig. S4b, Table S5).

The strongest association, *TraesCS3B02G602200* (~ 822.2 Mbp), linked with our peak markers (*wsnp_Ex_c13284_20948460* and *BS00091257_51*), showed consistent pleiotropic effects on PD (Fig. 3a–c), SWW, PWW, SS, and PS across three environments (Fig. 4a–c, Table S4). Five of the 12 *SSt1*-associated SNP markers were previously linked to stem solidity and pith thickness (Table S7). Additionally, five of the 11 putative genes identified within the interval, including our peak association, were reported by Zhao (2019) as regulators of pith thickness (Table S7). The remaining gene hits, including *TraesCS3B02G596700*, TraesCS3B02G597500, and *TraesCS3B02G603400* (Table S5), have not been previously associated with stem or peduncle traits and may represent novel components of this regulatory module.

The antagonistic allelic effects at five markers (Figs. 3d–f, 4d–f, Table S4) revealed a clear genetic trade-off between stem diameter and solidness. This antagonism was reflected in consistently negative genomic and phenotypic correlations between SD and SS as well as between PD and PS (r_*g*_ = − 0.51 to − 0.58; r_*p*_ = − 0.47 to − 0.52) across the three environments (Fig. 2, Table S3). All five markers showing opposing allelic effects reside in strong linkage disequilibrium (Fig. 3g), indicating either a single pleiotropic allele effect or a closely clustered causal interval rather than independent loci.

Our findings confirm and expand the functional scope of the *SSt1* region beyond stem solidness to include stem and peduncle diameter and demonstrate a reproducible genetic trade-off between these traits. Our results indicate that the *SSt1* region harbors one or more loci that jointly modulate multiple stem traits under Mediterranean terminal drought, with *TdDof* representing the solidness and other gene hits in the *SSt1* region contributing additional stem architectural effects. Whether the observed pleiotropic effect arises from a single gene, several tightly linked genes, or regulatory interactions involving *TdDof* and nearby gene hits cannot be determined by GWAS alone and will require fine-mapping and functional characterization.

A secondary MTA on chr. 3D (607.9–612.9 Mbp), explained 13.58–33.81% of the variation in PWW and PS (Tables S4 and S5), which is in accordance with the already identified locus on chr. 3D for stem solidness (*Qss.msub-3DL*; 31% PVE; Lanning et al. 2006), and stem wall thickness (*Qswt-3D*; 7% PVE; Niu et al. 2022), and reinforces the critical role of homoeologous group 3 in shaping stem architecture.

It is important to note that gene hit identification was based on a positional, SNP-centered approach reporting gene hits that directly overlapped or immediately flanked significant SNP in the RefSeq v1.1 assembly. Although this approach ensures a clear physical link between SNPs and gene hits, it does not include all genes within the broader LD blocks surrounding each MTA. Future work should extend gene hits extraction across the entire LD interval and apply fine-mapping to refine these regions.

### GWAS-identified environment-specific loci for peduncle length and stem biomass allocation under the Mediterranean climate

Peduncle length is a principal component of plant height and a key conduit of assimilate transport to developing grain. In the present study, five environment-specific SNPs associated with PL (4–6.7% PVE) were detected on chr. 6A under TD, 2B under WW, and 5B, 6B, 7B under RF (Fig. S3, Tables S4 and S5), indicating distinct genetic regulation across different moisture regimes.

The small individual effects and environment-dependent expression of these loci align with recent GWAS and QTL studies (see Table S7). Liu et al. (2023) identified *QTL-6D.*1 (13.6–24.2% PVE), a major PL QTL that showed an additive effect with the semi-dwarfing reduced height (*Rht*) alleles (*Rht-B1b* and *Rht-D1b*) in reducing both plant height and PL. In this study, no PL-associated marker co-localized with the known *Rht*-conferring locus, indicating that the environment-specific peduncle length loci identified here represent novel regions independent of the major *Rht* loci; however, they exhibit moderate control.

Stem and PDW are direct indicators of structural biomass investment and of the remobilization of assimilates to developing grains. In this study, 17 environment-specific SNPs and gene hits were associated with stem and PDW (Tables S4 and S5). Few studies have directly examined the genetic control of stem or PDW. Nouraei et al. (2024) identified 14 markers (Table S7) associated with shoot dry weight under drought and well-watered conditions, with several loci showing significant environment-by-treatment interactions, indicating the polygenic and environment-responsive nature of stem biomass traits.

Given that PL, stem, and PDW are governed by many small-effect QTLs, genomic selection models that capture additive effects across loci are likely a more effective breeding strategy than single marker selection for these traits. The positive correlations between stem biomass traits and TGW and GYPS make them valuable targets for selection to improve drought adaptation. However, phenotypic selection should not be overlooked, particularly non-destructive high-throughput phenotyping (HTP) proxies that are scalable in the field.

### Integration of genotype × environment interactions improved the accuracy of stem traits genomic prediction

One of the major challenges in genomic prediction for multi-environment data is predicting the performance of new or existing lines in both novel and familiar environments. We used multi-environment data to assess the PAs of stem traits, accounting for *M* × *E*, with SE environment models as a reference for comparing various multi-environment CV scenarios (Fig. S2). Our results showed that single-environment PAs were highly trait- and environment-specific. For instance, the PA for PL ranged from 0.47 to 0.61 across environments, while PDW and PWW exhibited minimal PA (0.09 and 0.14, respectively, under rain-fed conditions; Fig. 5a). The SE models treat each environment as independent and fail to separate broad adaptation from specific environmental noise, explaining why breeding programs focusing on a single location may fail to identify truly superior genotypes (Crossa et al. 2016).

In contrast, ME genomic prediction models significantly improved PAs across all CV scenarios (Fig. 5b–d). In the current study, the ME model increased the PAs by 35% (CV0- predicting known lines in an unseen environment), 9% (CV1- predicting new lines in a familiar environment), and 46.5% (CV2- predicting lines in a partly known environment) compared to the SE model (Table S6). Tomar et al. (2021) found similar results in spring wheat, with ME models providing 2–3 times higher PAs than SE. The ME models divide marker effects into two types: stable across environments (main effects) and those that are specific to each environment, enabling more reliable selection (Lopez-Cruz et al. 2015).

The CV0 and CV2, which simulate practical breeding by predicting phenotypes in environments with completely unobserved or partly known data, demonstrate the transformative power of *G* × *E* interaction modeling. Traits with _bs_*h*^2^ (PWW, PS, PL, PD) showed substantial improvement. For example, PS (_bs_*h*^2^ = 0.93) achieved PAs of 0.83–0.90 in CV0 and 0.76–0.82 in CV2 compared to PAs of 0.17–0.30 in SE, representing an increase of 66–80% and 63–78%, respectively (Fig. 5b, d, Table S6). These results are consistent with Sadeh et al. (2024), who showed that multi-environment models outperform the SE model for yield-related traits, with broader evidence supporting this.

Incorporating *G* × *E* effects improves PA by 36–57% compared to models without it (Tomar et al. 2021; Robert et al. 2022), and up to 49% when environmental covariates were integrated (Montesinos-López et al. 2024). The improvement in CV0 and CV2 highlights the effectiveness of a sparse testing design for predicting unobserved environments or partially phenotyped lines. By borrowing information across environments and modeling marker × environment interactions, it recovers genetic signals lost in SE analysis, helping breeders to identify genotypes with stable performance across diverse environmental conditions, rather than those suited to a specific environment (Sukumaran et al. 2017; Jarquín et al. 2017; Cuevas et al. 2017). Collectively, our results support integrating ME genomic selection models that incorporate *G* × *E* interactions to improve stem traits in Mediterranean wheat breeding programs.

## Conclusion and future perspective

Our study provides a high-resolution dissection of the genetic architecture of wheat stem traits under Mediterranean conditions. We reject our initial hypothesis of a trade-off between stem biomass investment and grain yield. Within semi-dwarf spring wheat grown at conventional sowing density (220 seeds per m^2^) under Mediterranean conditions, we find no such trade-off. Instead, we demonstrate that increased internode diameter, dry weight, and peduncle length are critical for sustaining grain size and yield per spike under terminal drought. This indicates that robust stem architecture enhances source strength without inherently penalizing yield.

A pivotal finding is the antagonistic pleiotropy at the *SSt1* locus (chr. 3B), where an increase in stem solidness constrains increases in stem diameter. This genetic "balancing" mechanism explains the difficulty in simultaneously optimizing these traits and underscores the necessity of selecting for environment-specific "stem ideotypes". Furthermore, by accounting for *M* × *E* interactions, we achieved a 46.5% increase in genomic prediction accuracy, highlighting the power of multi-environment frameworks in climate-resilient breeding. Notably, the prediction models were trained and validated within the same panel; thus, a future study is needed to provide independent validation under similar Mediterranean conditions. Ultimately, integrating GWAS-identified pleiotropic hubs such as *SSt1* with *G* × *E*-aware selection models offers a robust roadmap for developing the next generation of wheat cultivars capable of maintaining food security in a fluctuating climate.

## Supplementary Information

Below is the link to the electronic supplementary material.

Supplementary file1 (XLSX 111 KB)

Supplementary file2 (PDF 730 KB)

## Data Availability

The datasets used and analyzed during the current study are available from the corresponding authors upon reasonable request.
