# Supplementary material for: Antagonistic pleiotropy at the stem solidness 1 locus balances wheat stem architecture and yield components under terminal drought
Source: Theor Appl Genet. 2026 Aug 16;139(9):230. doi: 10.1007/s00122-026-05330-6 (PMC13478034; doi:10.1007/s00122-026-05330-6)
Supplement: Supplementary file 1 [file 122_2026_5330_MOESM2_ESM.pdf]

Antagonistic pleiotropy at the *stem solidness 1* locus balances wheat stem architecture and yield components under terminal drought

Supplementary Figures

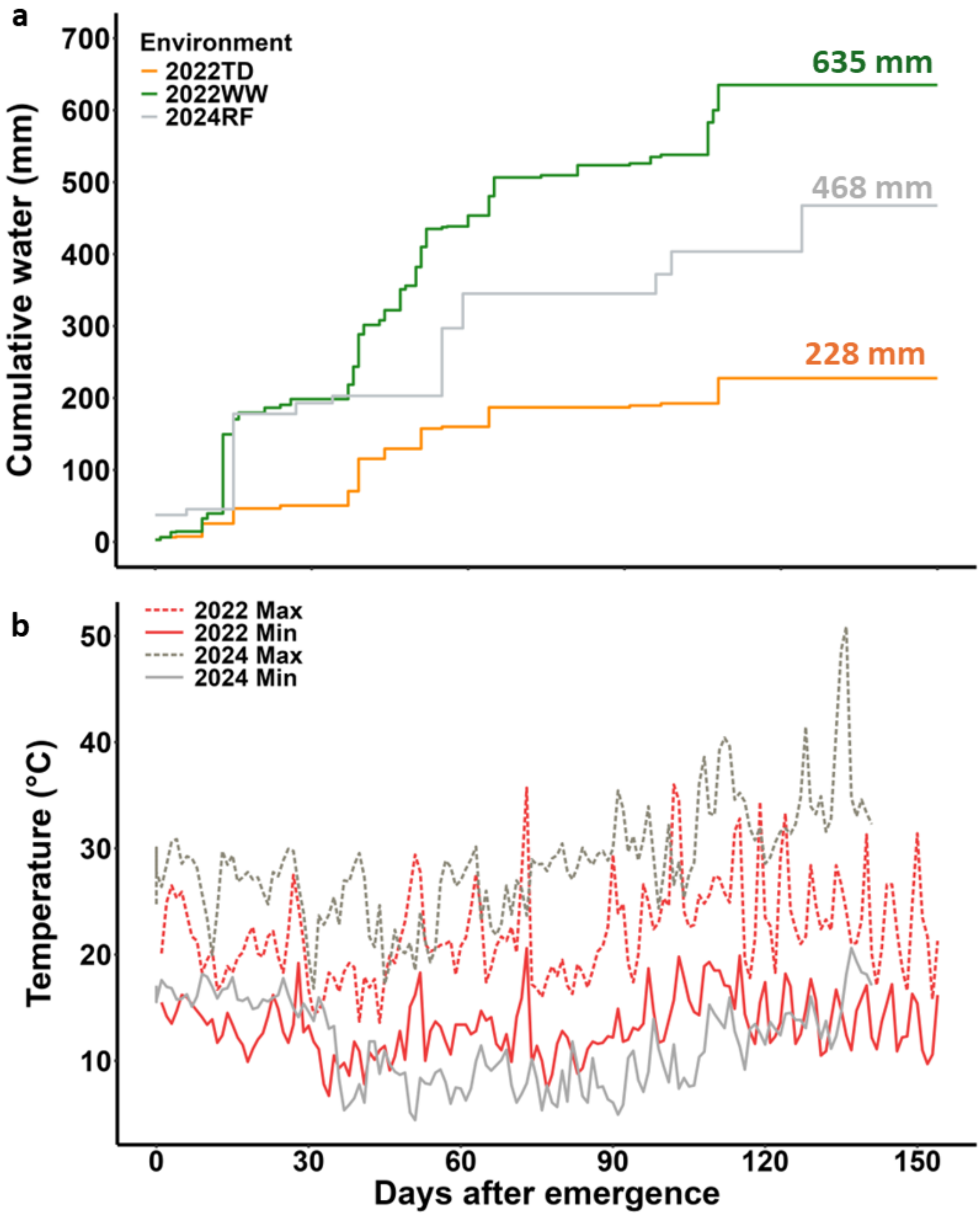

Figure S1. Environmental conditions in the three environments: Well-watered (2022WW), terminal drought (2022TD), and rain-fed (2024RF). (**a**) Cumulative water (precipitation and irrigation), and (**b**) seasonal minimum and maximum temperature.

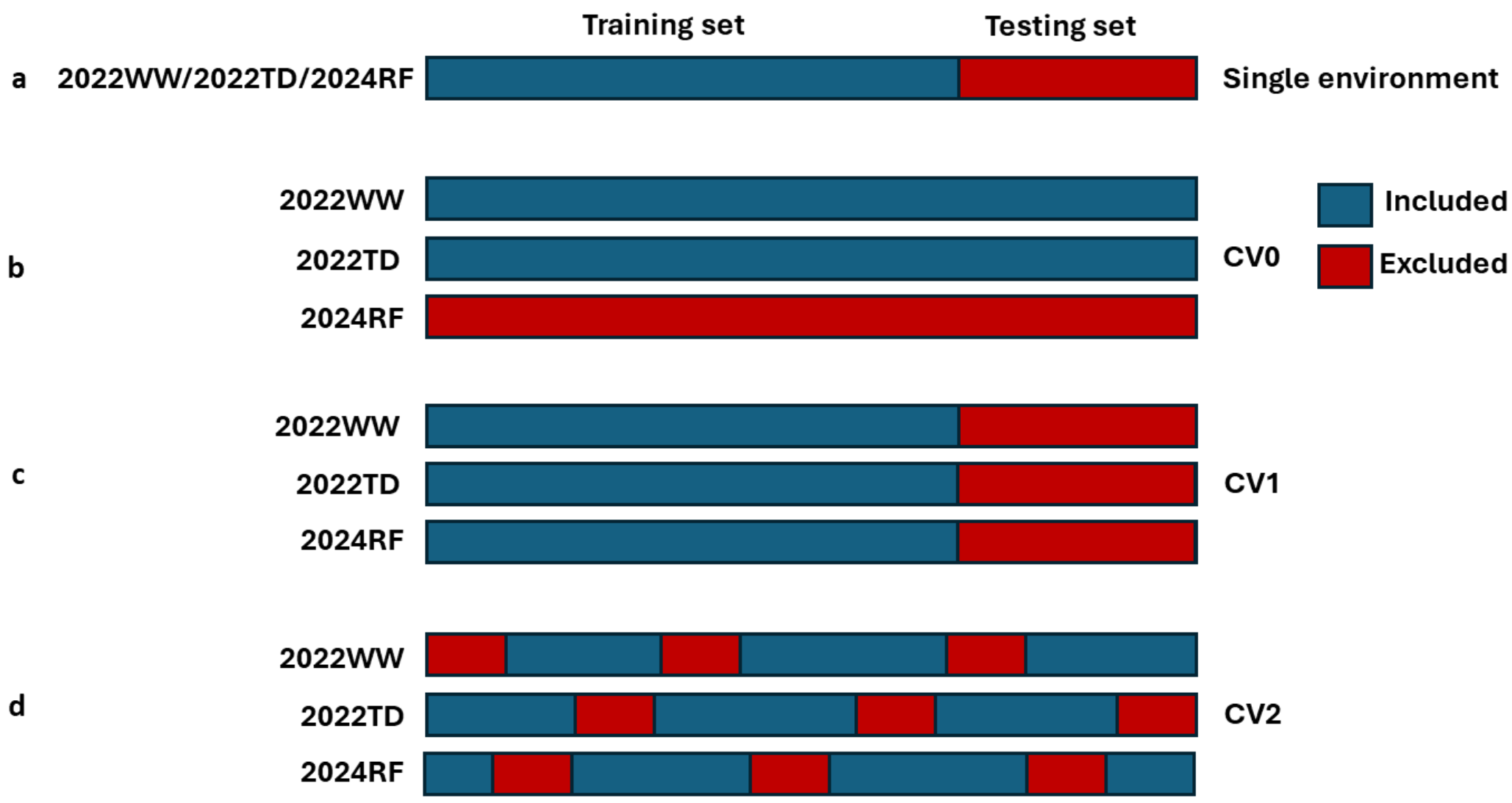

Figure S2. Single-and multi-environment genomic prediction cross-validation scenarios. (**a**) Single-environment analysis; (**b**) All the lines in two environments were used to predict the same lines in a new environment (CV0), (**c**) Performance of new lines in a known environment (CV1), and (**d**) Predict lines that were evaluated in only one environment (CV2). The environments are: Well-watered (2022WW), terminal drought (2022TD), and rain-fed (2024RF).

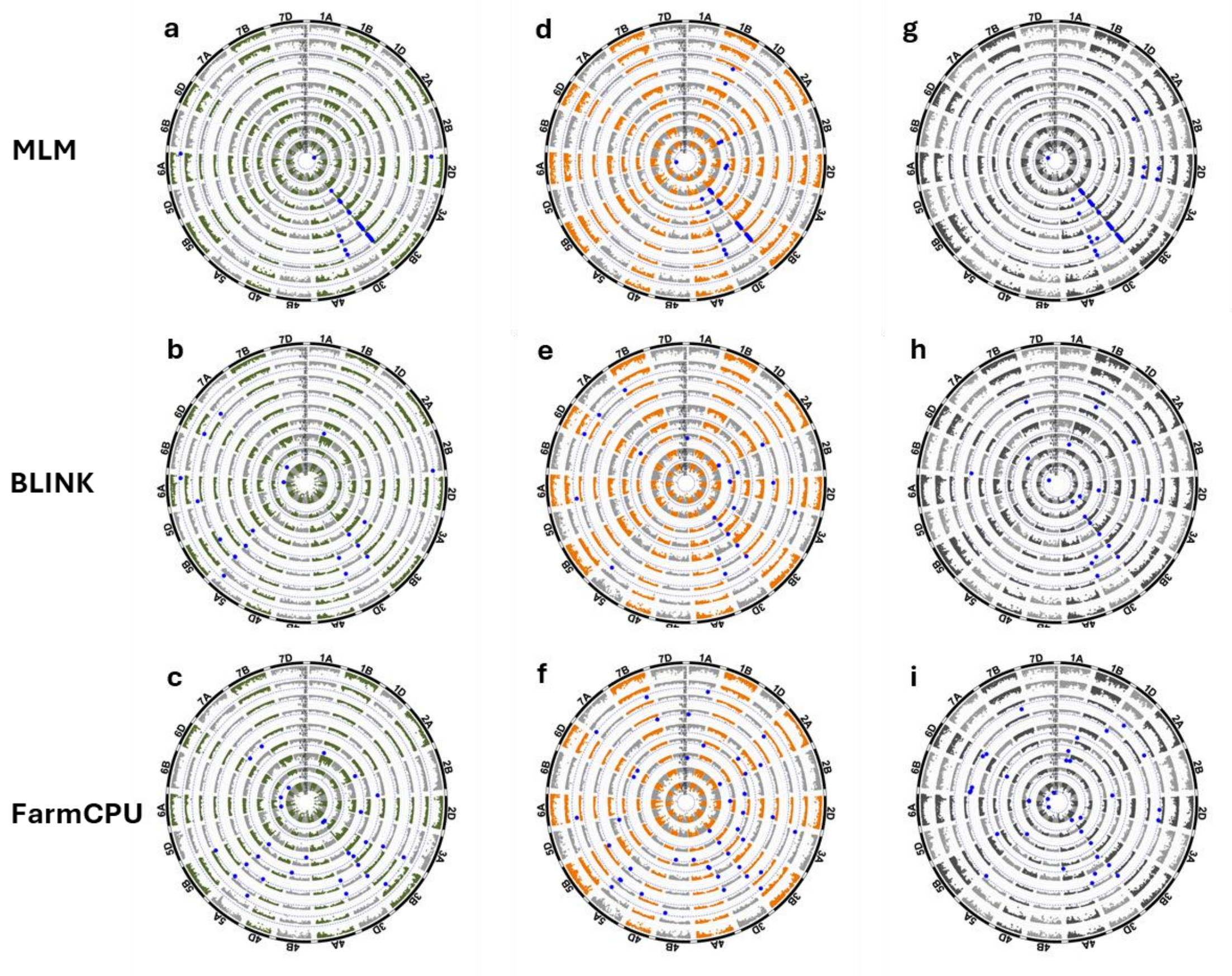

Figure S3. Genetic architecture of stem traits and yield components. Circular Manhattan plots for the (from inner to outer circle): peduncle length, stem diameter, stem wall width, stem solidness, peduncle diameter, peduncle wall width, peduncle solidness, stem dry weight, and peduncle dry weight under (**a-c**) well-watered, (**d-f**) terminal drought, and (**g-i**) rain-fed using: Mixed linear model (MLM), Bayesian information and linkage disequilibrium iteratively nested keyway (BLINK), and Fixed and random model, circulating probability unification (FarmCPU). Dashed blue circular lines are significant thresholds, while the significant SNP markers are highlighted in blue.

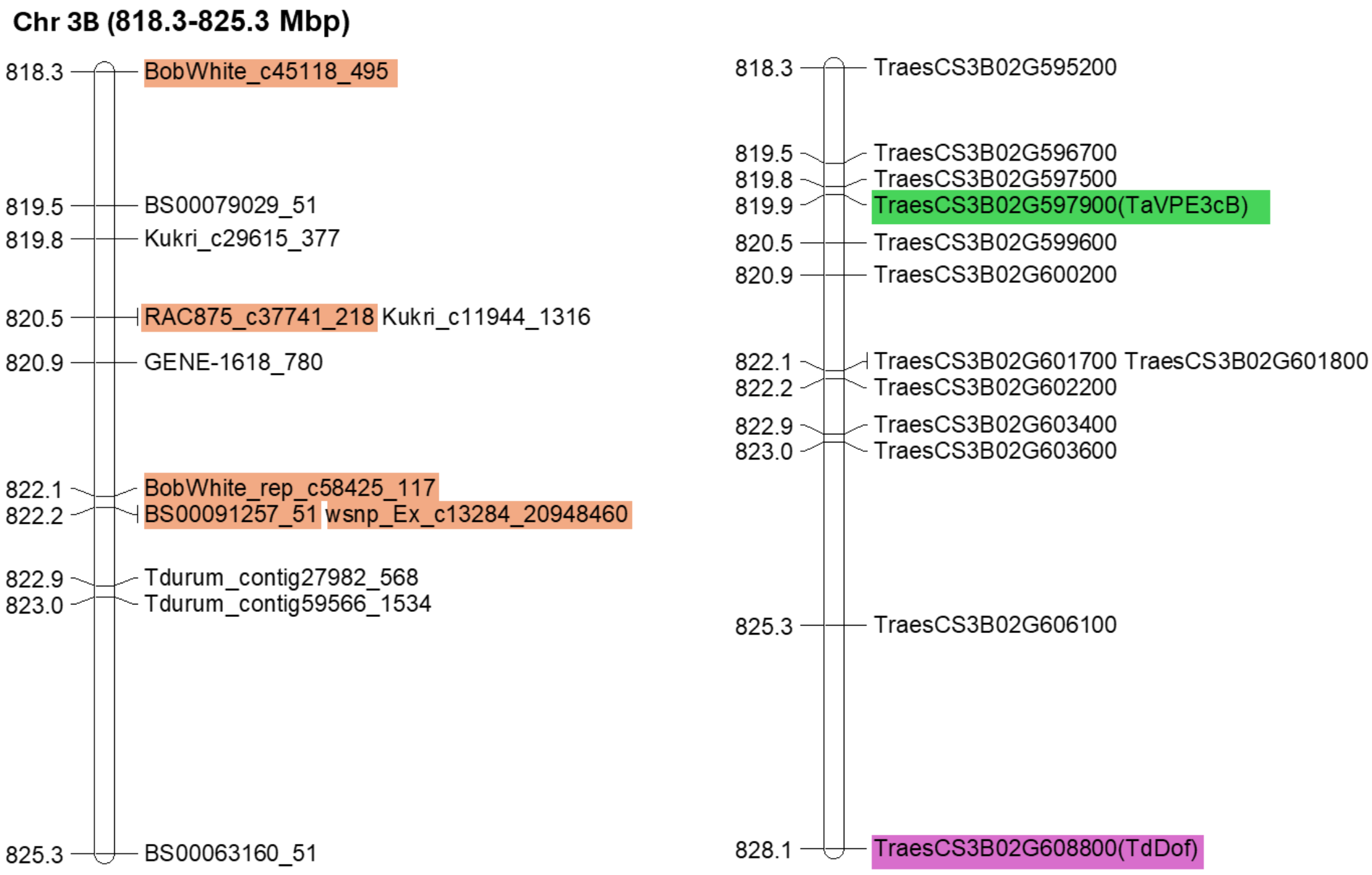

Figure S4. Positions of twelve significant markers spanning 818.2–825.3 Mbp showed associations with stem traits (stem and peduncle wall width and solidness), with peak signals clustering at 822 Mbp on chromosome 3B. Markers highlighted in orange (*BobWhite_c45118_495*, *wsnp_Ex_c13284_20948460*, *BS00091257_51*, *BobWhite_rep_c58425_117*, *RAC875_c37741_218*) exhibited antagonistic allelic effects between diameter and solidness. (**b**) Positions of gene hits associated with the significant markers (IWGSC RefSeq v1.1). The gene highlighted in purple was reported by Nilsen et al. (2020) to control stem solidity through copy number variation in bread wheat, while the gene in green was identified by Liu et al. (2023) as a candidate gene for stem pith thickness.

References

Liu Q, Zhao Y, Rahman S, et al. (2023) The putative vacuolar processing enzyme gene *TaVPE3cB* is a candidate gene for wheat stem pith-thickness. Theor Appl Genet 136:138. https://doi.org/10.1007/s00122-023-04372-4

Nilsen KT, Walkowiak S, Xiang D, et al. (2020) Copy number variation of *TdDof* controls solid-stemmed architecture in wheat. Proc Natl Acad Sci USA 117:28708-28718. https://doi.org/10.1073/pnas.2009418117
